# Global priorities for large-scale biomarker-based prospective cohorts

**DOI:** 10.1016/j.xgen.2022.100141

**Published:** 2022-06-08

**Authors:** Rory Collins, Mary K. Balaconis, Søren Brunak, Zhengming Chen, Mary De Silva, J. Michael Gaziano, Geoffrey S. Ginsburg, Prabhat Jha, Pablo Kuri, Andres Metspalu, Nicola Mulder, Neil Risch

**Affiliations:** 1 Nuffield Department of Population Health, University of Oxford, Oxford, UK; 2 Broad Institute of MIT and Harvard, Cambridge, MA, USA; 3 Novo Nordisk Foundation Center for Protein Research, Faculty of Health and Medical Sciences, University of Copenhagen, Copenhagen, Denmark; 4 Clinical Trial Service Unit & Epidemiological Studies Unit and Medical Research Council Population Health Research Unit, Nuffield Department of Population Health, University of Oxford, Oxford, UK; 5 Head of Population Health, Wellcome Trust, London, UK; 6 Department of Medicine, Division of Aging, Brigham and Women’s Hospital and the VA Boston Healthcare System, Harvard Medical School, Boston, MA, USA; 7 All of Us Research Program, National Institutes of Health, Bethesda, MD, USA; 8 Dalla Lana School of Public Health, Centre for Global Health Research, St. Michael’s Hospital, University of Toronto, Toronto, ON, Canada; 9 Instituto Tecnológico de Estudios Superiores de Monterrey, Monterrey, Mexico; 10 Estonian Genome Center, Institute of Genomics, Estonian Biobank, University of Tartu, Tartu, Estonia; 11 Department of Integrative Biomedical Sciences, Computational Biology Division, IDM, CIDRI-Africa WT Centre, University of Cape Town, Cape Town, South Africa; 12 Institute for Human Genetics, University of California, San Francisco, CA, USA

## Abstract

The focus of this paper is on strategic approaches for establishing population-based prospective cohorts that collect and store biological samples from very large numbers of participants to help identify the determinants of common health outcomes. In particular, it aims to address key issues related to investigation of genetic, as well as social, environmental, and ancestral, diversity; generation of detailed genetic and other types of assay data; collection of detailed lifestyle and environmental exposure information; follow-up and characterization of incident health outcomes; and overcoming obstacles to data sharing and access (including capacity building). It concludes that there is a need for strategic planning at an international level (rather than the current *ad hoc* approach) toward the development of a carefully selected set of deeply characterized large-scale prospective cohorts that are readily accessible by researchers around the world.

## Teaser

Collins et al. highlight the value of prospective cohort studies that involve hundreds of thousands of participants, who are followed for a prolonged period, to investigate genetic, lifestyle, and environmental determinants of disease. They discuss the need for strategic planning at an international level to develop a set of deeply characterized, informatively diverse, large-scale prospective cohorts that are readily accessible by researchers around the world.

## Main text

### Strategic need for large, long-term, prospective cohorts in different settings

Prospective cohort studies in which individuals from some particular population are assessed in detail at the start of the study (ideally with stored bio-samples), with their health then followed for many years to identify well-characterized incident cases of disease, allow the relevance of both genetic and non-genetic risk factors (and of the interactions between them) for many different conditions to be investigated in the same setting. However, depending on the duration of follow-up, only a relatively small proportion of the participants in a prospective study are likely to develop any particular condition, even one that is relatively common. Consequently, prospective studies need to involve large numbers of participants (e.g., hundreds of thousands) followed for a prolonged period of time so that sufficiently large numbers of cases of any particular disease become available to support reliable investigation of its determinants.

Prospective cohort studies avoid many of the biases inherent to retrospective case-control studies in which individuals who have developed some particular disease are compared with control individuals without that disease, but some potential biases still need to be considered in their analysis and interpretation. For example, prodromal disease may affect the values of risk factors recorded at the start of a prospective study, although the effects of such reverse causation can usually be minimized by restricting analyses to individuals without particular diseases at the start or by excluding events that occur during the first few years of follow-up (or even longer for some conditions, such as chronic lung disease or dementia). Non-representative sampling may lead to selection bias if prognostic variables influence participation, although the magnitude of such effects may well be small by comparison with other potential sources of bias, as well as with the random errors due to a lack of sufficient numbers of cases of the diseases of interest. That is, both systematic errors due to biases and random errors due to too few cases need to be considered equally seriously.

Large prospective cohorts with stored biological samples and prolonged follow-up involve a costly long-term commitment, so planning new studies should involve careful consideration of the specific evidence gaps that they would fill. It should be noted that, whereas associations of risk factors with disease that are identified within a particular study should be enduring (subject to the range of risk factor levels and disease rates studied: Box 1), the risk factor levels and disease rates within the population studied are likely to change over time and differ from those found in other populations. Consequently, what is required is not that these prospective cohorts are representative of any particular population, but instead that they provide generalizable information about the full range of risk factor levels for many different diseases.

Box 1 **Strategic needs for biospecimen-based prospective cohorts**

**Scale:** reliable assessment of associations with risk factors requires studies in which many thousands of incident cases of each health outcome of interest are recorded.

**Range:** evaluation of the full range of internal and external exposures, and of rates of different diseases, requires studies to be established in some carefully selected populations.

**Depth:** detailed characterization of participants in carefully selected existing, as well as new, cohorts would allow more comprehensive investigation of risk factor associations.

**Follow-up:** the ability to follow the health of participants long term, and to characterize their health outcomes fully, is required for sensitive and specific disease associations.

**Quality:** data on health-related exposures and disease outcomes that are complete and of high quality facilitate the reliable investigation of the determinants of disease.

In order that the prospective cohorts established around the world are able collectively to provide widely generalizable information about associations between risk factors and health outcomes, there is a need to ensure that sufficiently large numbers of participants are included to cover the full range of many different internal (e.g., genetic and ethnic) and external (e.g., lifestyle and environment) exposures. Diversity in all its aspects is an important value in biomedical research. Much attention has been drawn to the lack of racial, ethnic, and ancestral diversity in genetic studies, in particular genome-wide association studies.^1^ Existing cohorts may include greater ethnic and socio-economic diversity than is often considered to be the case (for example, people of African ancestry in some United States-based cohorts). Even so, there is a need to establish additional cohorts in carefully selected settings in different parts of the world in order to support investigation of the full range of all types of diversity of risk factors. Moreover, rates of most health outcomes differ hugely between different populations, and this diversity should also be encompassed by different studies.^2^^,^^3^ The ability to investigate the associations of a wide range of different exposures with a wide range of different disease rates is likely to generate widely generalizable findings (for example, rare genetic variants may identify novel therapeutic targets; risk factor levels below the “normal” range in a population may yield novel preventative strategies).

The SARS-CoV-2 pandemic has highlighted the value of having large prospective cohorts with detailed information about exposures (including genetic data and stored biological samples), combined with ongoing follow-up of their health outcomes, to help respond rapidly to health crises. For example, such studies have allowed researchers to identify those individuals at particular risk of a bad outcome from SARS-CoV-2, which has helped in the development of strategies for protecting vulnerable individuals.^4^, ^5^, ^6^ They have also provided efficient structures for adding specific information (e.g., cohort-wide antibody testing) that allows researchers to study longer-term effects of infections with different severities on health outcomes.^7^^,^^8^ In developing strategies for improved pandemic preparedness in the future, prospective cohorts in different populations will have an important role to play.

### Value of detailed characterization of risk exposures and health outcomes

As well as ensuring that large-scale prospective cohorts are established that involve a sufficiently wide range of exposures, it is important to consider how to ensure that each such study involves sufficiently detailed characterization of the participants to allow the full range of exposures to be studied. Consequently, a strategy of investing not only in establishing additional cohorts in carefully selected settings but also in enhancing the characterization of selected cohorts is likely to be of greatest value for global research (assuming the data would be made widely available to researchers; Box 1) and the populations that are studied. Such enhanced characterization might include collection of different types of biological sample (e.g., blood, urine, and stool) that can support many different types of assays (e.g., genetic, proteomic, metabolomic, and metagenomic); comprehensive assessment of previous lifestyle, environment, and health; extensive range of anthropometric, physical, and psychological measures; and detailed imaging and remote monitoring data. The addition of data on environmental exposures (e.g., direct assessment at the level of individuals; linkage to geo-spatial data at area level) would enhance understanding of their relevance to incident disease.

The recent focus in large retrospective and prospective studies has been on genetic determinants of disease, chiefly as a consequence of the technological advances that have been made in increased throughput and reduced cost of genetic assays (initially for genotyping but now for large-scale exome and genome sequencing).^9^^,^^10^ As a consequence of this ability to conduct genetic assays at a very large scale, there has been an explosion of information about associations of genetic variation with both risk factors for disease and with many different health outcomes.^11^ The opportunity is now arising to generate similar large-scale information about proteomic, metabolomic, and other -omic measures, which will be of value for elucidating causal pathways. As has been the case with genetics, it seems likely that different approaches will be used (i.e., different types of assay platforms), each of which provides complementary information. In considering assays beyond genetics, it is important to recognize that (by contrast with the genome) values in these other -omic domains are likely to vary considerably over time within individuals. Consequently, a potentially valuable enhancement of participant characterization in prospective studies would involve repeated assessment of the whole cohort during follow-up (including collection of biological samples and, indeed, other types of non-genetic exposure, such as lifestyle and environment) in order that the impact of changes over time on disease trajectories can be assessed comprehensively.

In contrast to retrospective case-control studies, which start with well-characterized health outcomes, prospective cohorts start with very large numbers of people who are characterized at baseline and then need to have their health followed reliably for many years in order to determine which of them develop any particular condition. Serious concerns in prospective cohorts are participant retention, losses to follow-up, and continuity of information on disease onset. Missing health outcomes that occur during follow-up is not likely to produce material bias in the estimates of associations of risk factors with disease (unless retention is related to both the risk factor and the disease outcome), but it will reduce statistical power to detect associations.^12^ Consequently, in choosing where to establish prospective cohorts, it is prudent to ensure that secure long-term follow-up of the health of the participants can be readily maintained. Inaccurate attribution of health outcomes or lack of specificity (e.g., not being able to separate ischemic and hemorrhagic strokes) may well lead to materially biased estimates of associations. Consequently, there would be value in obtaining supplementary information specific to particular health outcomes from medical records (e.g., histology, imaging), as well as by using novel tools (e.g., remote assessments via mobile phones and other internet-enabled devices) and implementing advanced analytic tools (e.g., artificial-intelligence-assisted data mining), to enhance both the accuracy and the specificity of diagnoses in ways that are scalable to large numbers of different types of health outcome.

### Future priority setting: Incentives for researchers and communities

Access to detailed data about very large numbers of participants from a limited number of prospective cohorts in carefully selected settings, with detailed information about the health outcomes occurring during prolonged follow-up, is likely to be of greater value for international research than a larger number of less well-characterized cohorts. The International HundredK + Cohorts Consortium (IHCC) Global Cohorts Atlas (https://atlas.ihccglobal.org/)^13^ can help to identify both those cohorts that are likely to provide the greatest return on further investment in their enhancement and those settings in which additional cohorts might best fill gaps in the range of risk factors and health outcomes that can be studied (i.e., in order to address all forms of diversity). Previous experience in setting up cohorts in different settings can help to ensure that any new cohorts are established in ways that increase the likelihood of their successful recruitment and long-term follow-up.^14^ Emphasizing the utility of the findings that emerge from such cohorts for the population in which they are embedded may encourage engagement from national governments, health care providers, and the wider public, facilitating their long-term maintenance. All of these factors are likely to be critical to ensuring the financial and operational (e.g., field work, sample storage, etc.) sustainability of such studies, and new funding strategies should also be considered (including the involvement of diverse types of financial institution and private companies interested in the potential for enabling important discoveries).

There is also a need to consider how best to incentivize the scientists who do the work to develop and maintain such resources (i.e., what is a reasonable academic return for them) especially in resource-poor settings where analytic capacity is often limited. This is likely to require some periods of protected access for the creators of cohorts, as well as for other local researchers, in order to maximize benefits for the local community. In addition, funders should consider how best to invest in developing research capacity in such settings (e.g., core support, training opportunities) to enhance the ability of local researchers and to develop the “cohort leaders of tomorrow.” Efforts should also be made to explain the value of such studies for improving health nationally to governments and the wider public. The development of technical solutions (e.g., locally hosted cloud-based platforms that allow use of data to be monitored) may help to address concerns about allowing access by external researchers to genetic and other biomedical data (such as the restrictions on use that exist explicitly in India and China, and implicitly in some other locations), as well as facilitating collaboration and cooperation between different cohorts.^15^ In addition, such approaches may make it possible for researchers with limited local computing facilities not only to access but also to analyze these increasingly large prospective cohort datasets provided they are given sufficient guidance and support as the data become increasingly complex.

In conclusion, large prospective cohorts with stored biological specimens may well transform our understanding of the causes, prevention, and treatment of disease globally. For example, infections that are common in many low- and middle-income countries (such as malaria and tuberculosis) may have associated genetic and environmental risk factors (other than the relevant pathogen) that make infection or progression to disease more probable. Likewise, studies of risk factors for chronic diseases that are common in high-income countries may become increasingly relevant to developing countries as lifestyles in their populations change. However, given the financial and organizational cost of establishing, maintaining, and enhancing large prospective cohorts, a globally strategic approach to their establishment is required. Such an approach would help to ensure that these cohorts are sustainable and accessible by the international research community as a whole for the widest possible range of investigation into the genetic and non-genetic determinants of many different health conditions across informatively diverse populations.
